# Revisiting *Trypanosoma rangeli* Transmission Involving Susceptible and Non-Susceptible Hosts

**DOI:** 10.1371/journal.pone.0140575

**Published:** 2015-10-15

**Authors:** Luciana de Lima Ferreira, Marcos Horácio Pereira, Alessandra Aparecida Guarneri

**Affiliations:** 1 Vector Behavior and Pathogen Interaction Group, Centro de Pesquisas René Rachou, Fundação Oswaldo Cruz, Belo Horizonte, Brazil; 2 Departamento de Parasitologia, Instituto de Ciências Biológicas da Universidade Federal de Minas Gerais, Belo Horizonte, Brazil; Universidade Federal do Rio de Janeiro, BRAZIL

## Abstract

*Trypanosoma rangeli* infects several triatomine and mammal species in South America. Its transmission is known to occur when a healthy insect feeds on an infected mammal or when an infected insect bites a healthy mammal. In the present study we evaluated the classic way of *T*. *rangeli* transmission started by the bite of a single infected triatomine, as well as alternative ways of circulation of this parasite among invertebrate hosts. The number of metacyclic trypomastigotes eliminated from salivary glands during a blood meal was quantified for unfed and recently fed nymphs. The quantification showed that ~50,000 parasites can be liberated during a single blood meal. The transmission of *T*. *rangeli* from mice to *R*. *prolixus* was evaluated using infections started through the bite of a single infected nymph. The mice that served as the blood source for single infected nymphs showed a high percentage of infection and efficiently transmitted the infection to new insects. Parasites were recovered by xenodiagnosis in insects fed on mice with infections that lasted approximately four months. Hemolymphagy and co-feeding were tested to evaluate insect-insect *T*. *rangeli* transmission. *T*. *rangeli* was not transmitted during hemolymphagy. However, insects that had co-fed on mice with infected conspecifics exhibited infection rates of approximately 80%. Surprisingly, 16% of the recipient nymphs became infected when pigeons were used as hosts. Our results show that *T*. *rangeli* is efficiently transmitted between the evaluated hosts. Not only are the insect-mouse-insect transmission rates high, but parasites can also be transmitted between insects while co-feeding on a living host. We show for the first time that birds can be part of the *T*. *rangeli* transmission cycle as we proved that insect-insect transmission is feasible during a co-feeding on these hosts.

## Introduction

*Trypanosoma rangeli* was first described by Tejera in 1920 [1]; since then, many studies have been undertaken to examine its interactions with its hosts. In the Americas, this parasite infects triatomines and mammals, including man, although it does not cause disease in humans [2]. However, *T*. *rangeli* exhibits different levels of pathogenicity to its invertebrate hosts [3–6]. Parasite development inside vectors begins when trypomastigote forms are ingested together with blood meals from infected mammals. These forms transform into epimastigotes inside the vector midgut and then multiply and colonize the intestinal tract. During the infection, some parasites can cross the intestinal epithelium and reach the hemocoel. Parasites that manage to cross the hemolymph divide and migrate to the salivary glands where they transform into metacyclic trypomastigotes, which are the forms that infect vertebrate hosts and that are transmitted in the saliva during blood meals [2].

Despite our understanding of the transmission process, gaps remain in our understanding of the triatomine-parasite-mammal interaction; the most intriguing questions concern the development of *T*. *rangeli* in vertebrate hosts. To date, it remains unclear how the parasite is maintained in these hosts due to conflicting findings [7–9]. Apparently, no intracellular replicative forms are present, as is observed in *Trypanosoma cruzi* [10] and *Leishmania* spp. [11], and no multiplicative forms are present in the blood circulation, as occurs in *T*. *brucei* [12] and *T*. *conorhini* [13]. Nevertheless, *T*. *rangeli* is found abundantly in nature [14, 15] and infects several triatomine species and an extensive variety of mammals [16–18]. In addition, *T*. *rangeli* completes its development only in triatomines from the genus *Rhodnius* [15]. Biological and molecular studies suggest that these parasites and their vectors have coexisted over a long period, with diverging lineages that are associated with sympatric species of *Rhodnius* but without an apparent association with vertebrate host species [18–20]. This evolutionary relationship between *T*. *rangeli* and its invertebrate hosts, in conjunction with the lack of reports of parasite multiplication in vertebrate hosts, suggests the existence of forms of transmission other than the classic form in which insects become infected while feeding on an infected mammal. There are a few reports that suggest that insect-insect parasite transmission can occur through hemolymphagy, when one insect takes hemolymph from a conspecific [21] or during a simultaneous blood feeding [22], although the relevance of these alternative transmission routes in parasite circulation has not been established. Therefore, we used a strain of *T*. *rangeli* that was maintained through cyclical passages in triatomines and mice and established a protocol that mimics the natural transmission to evaluate how *T*. *rangeli* circulates between mice and *Rhodnius prolixus* and the importance of insect-insect transmission for parasite circulation.

## Materials and Methods

### Organisms

The CHOACHI strain, which was isolated from naturally infected *R*. *prolixus* from Colombia [23], was used. Epimastigote forms were cultured at 27°C by two weekly passages in liver-infusion tryptose (LIT) supplemented with 15% fetal bovine serum (FBS), 100 μl of streptomycin/ml and 100 units/ml of penicillin. To maintain strain infectivity, parasites were submitted to triatomine-mouse passages every three months [24].

*Rhodnius prolixus* was obtained from a colony that was originally established with insects collected in Honduras in approximately 1990, which is maintained by the Vector Behavior and Pathogen Interaction Group. Insects were reared at 26±1°C and a relative humidity of 65±10% under natural illumination. The insects were fed on chicken and mice that were anesthetized with intraperitoneal injections of a ketamine (150 mg/kg; Cristália, Brazil)/xylazine (10 mg/kg; Bayer, Brazil) mixture. All animal protocols followed the norms of FIOCRUZ regarding animal maintenance and experimentation and were approved by the Ethics Committee on Animal Use (Comissão de Ética no Uso de Animais—Fundação Oswaldo Cruz; CEUA-FIOCRUZ) under the number L-058/08.

Male mice from the Swiss Webster lineage weighing approximately 30 g and adult pigeons (*Columba livia*) of both sexes weighing approximately 450 g (both the mice and pigeons were obtained from the biotery of Centro de Pesquisa René Rachou) were used as vertebrate host models. The animals were anesthetized as described above. Rabbit blood was obtained from CECAL (Centro de Criação de Animais de Laboratório)–FIOCRUZ.

### *Rhodnius prolixus* infection

Third instar nymphs (7 days after molting) were fed on an artificial feeding apparatus containing citrated, heat-inactivated (56°C/30 min) rabbit blood containing a suspension of *T*. *rangeli* epimastigotes (1x10^5^ parasites/ml). Epimastigotes were obtained from a 10-day culture medium; the parasites were washed and resuspended in sterile phosphate buffered saline (PBS; 0.15 M NaCl in 0.01 M sodium phosphate, pH 7.4). To ensure the presence of parasites in the hemolymph and salivary glands, insects were also infected intracelomically as described previously [25]. Briefly, nymphs were inoculated in the side of the thorax with 1 μl of parasite suspension (5x10^4^ parasites/ml) after molting to the 4^th^ instar (7 days after molting). Twenty-four hours after inoculation, the nymphs were allowed to feed on anesthetized mice. Control insects were fed on rabbit blood and inoculated with PBS on the same days as the infected ones.

### Estimation of the number of parasites liberated by *T*. *rangeli*-infected nymphs during a blood meal on mice

The number of parasites liberated during a blood meal was estimated as previously described [26]. A group of nymphs was infected as described, and 45 days after intracelomic inoculation, the salivary glands from half of the nymphs (n = 11) were dissected. The remaining nymphs (n = 11) were fed on anesthetized mice, and their salivary glands were dissected immediately after completion of the blood meal. Each pair of glands was placed on a glass slide containing PBS to wash the glands and remove the hemolymph parasites. The glands were then transferred to 1.5-ml plastic microcentrifuge tubes containing 100 μl of PBS, ruptured with forceps, and the contents were homogenized. Ten microliters of the homogenate was collected and quantified using a Neubauer chamber. The remaining 90 μl was subjected to DNA extraction using the NucleoSpin Tissue XS kit (Macherey-Nagel, Bethlehem, PA, USA) according to the manufacturer's instructions. The purified DNA was diluted in 22 μl of water for subsequent estimation of the number of parasites using real-time PCR (qPCR). To quantify the number of parasites in the salivary glands of fed and unfed insects, a standard curve was constructed using serial dilutions of the plasmid pGEM-T Easy (Promega, Fitchburg, WI, USA) containing a 137-bp multicopy sequence related to the gene for RNA-Cl1 sno *T*. *rangeli*, which was used to detect the parasite (GenBank: AY028385.2, forward: 5′ gaaagcgcaagagagagat-3' and reverse: 5'-tgagatggctatcacgcaag-3') [27]. The plasmid was diluted to obtain a standard curve encompassing the range from 10^0^ to 10^7^ molecules; the resulting curve was used to estimate the number of copies present in each sample. The qPCR reactions were performed using an ABIPRISM 7500 Sequence Detection System (Applied Biosystems, Foster City, CA, USA). Each reaction was conducted in triplicate and contained 2 μl of DNA, 300 nM of each primer, and 12.5 μl Power SYBR Green PCR Master Mix (Applied Biosystems, Foster City, CA, USA) in a final volume of 25 μl. The DNA was amplified using the following protocol: 95°C for 10 min, followed by 40 cycles at 95°C for 15 sec and 60°C for 1 min. A melting curve analysis was used to verify that a single product was amplified. A negative control without the presence of primers was included. The number of parasites released from the salivary glands during the blood meal was estimated using a Neubauer chamber. The estimation was done by subtracting the average number of parasites present in the salivary glands after feeding from the average number of parasites present before feeding. PCR quantifications were used to estimate the percentage of reduction in the *T*. *rangeli* DNA amount in salivary glands from fed and unfed infected insects.

### *T*. *rangeli* transmission from infected mice to *R*. *prolixus* nymphs over time

Mice (n = 31, divided into three replicate experiments) were anesthetized and exposed to individual infected 5^th^ instar nymphs (30 days after molting) for 50 min. The fed nymphs were then removed, and the salivary glands were dissected to confirm infection (only mice that had been bitten by an infected nymph were used). On days 1–7, 15 and 30 after infection, the infected mice were anesthetized and offered to individual uninfected 5^th^ instar nymphs (30 days after molting) for 50 min. Five mice were also evaluated 126 days post infection (dpi). Immediately after feeding, the nymphs were transferred to a BOD chamber (27±1°C, 12:12 L:D) and maintained under these conditions for 21 days, after which the intestinal contents and hemolymph were examined for parasites. Insects that presented parasites in the hemolymph were maintained in the same conditions for 30 days, when the salivary glands were examined. At the end of each assay, all mice were tested for the presence of parasites by direct examination. Parasitemia was assessed by counting the trypomastigotes in 5 μl of tail vein blood [28]. Mice that were used as a food source for nymphs that proved negative were also bled at the end of the experiment (n = 3). The collected blood (~0.8 ml) was inoculated in LIT (1 ml) + NNN medium (1 ml), and the hemocultures were examined after 20 days.

### Insect-insect *T*. *rangeli* transmission during hemolymphagy

Hemolymphagy was evaluated as previously described [29]. Fifth instar nymphs containing parasites in their hemolymph served as the food source. Three days before the assay, infected nymphs were fed on anesthetized mice. To increase the feeding motivation of the starved insects, the infected 5^th^ instar nymphs were maintained at 40°C for three min immediately before the start of each assay. Nymphs were then individually immobilized with adhesive tape inside a cylindrical container (5.5 cm in diameter and 8.0 cm high) that was lined with filter paper. Second instar nymphs (10 days after molting; n = 10) were liberated and maintained in the container for 10 min together with the infected nymph. During this period, the number of nymphs that attempted to bite the fed nymph was recorded. This experiment was repeated twice. In a second experiment, 2^nd^ instar nymphs (20 days after molting; n = 10) were maintained together with one infected 5^th^ instar nymph (10 days post feeding) for 15 days in a cylindrical container (as described before) that was covered with cotton fabric. After this period, the 5^th^ instar nymph was removed, and the 2^nd^ instar nymphs were fed on anesthetized mice and transferred to a BOD chamber (27±1°C, 12:12 L:D). Insects were maintained under these conditions until molting to the 3^rd^ instar, when the intestinal tract, hemolymph and salivary glands were examined under microscopy to determine the presence of parasites.

### Insect-insect *T*. *rangeli* transmission by co-feeding on vertebrate

One donor-infected nymph and one recipient-uninfected nymph were placed into a cylindrical container (5.5 cm in diameter and 8.0 cm high) covered with cotton fabric and divided into two sectors by a narrow piece of cardstock paper, which prevented contact between the insects (n = 13 and n = 25 for mouse and pigeon, respectively). One anesthetized mouse or pigeon was placed ventral side down on the container for 40 min as a food source. After this, the vertebrate and the donor nymph were removed from the apparatus. The recipient nymph was transferred to a BOD chamber (27±1°C, 12:12 L:D) and maintained under these conditions for 21 days, after which the intestinal tract, hemolymph and salivary glands were examined to determine the presence of parasites. Additional assays were performed using five 5^th^ instar nymph recipients together with one 5^th^ instar donor for both mice (n = 4) and pigeon studies. In the experiment using pigeons as hosts, the following assays were performed: a) recipients feeding simultaneously with the donor, but placed 6 cm apart from it (n = 5 pigeons); b) recipients feeding 30 min after the donor had finished the meal (n = 5 pigeons); c) recipients feeding 24 h after the donor had finished the meal (n = 5 pigeons).

### Evaluation of complement-mediated *T*. *rangeli* lysis

*T*. *rangeli* lysis mediated by mice/pigeon complement action was evaluated as previously described [30]. Blood samples were obtained from anesthetized adult pigeons and mice. After incubation at 37°C for one hour, the blood was centrifuged for 5 min at 5,000 *g* and 4°C to obtain serum. Serum from each donor was divided into aliquots, and half of them were incubated at 56°C for 30 min to inactivate the complement system. All aliquots were stored at -70°C and each aliquot was used only once. Metacyclic trypomastigotes were obtained from salivary glands that were removed from infected 5^th^ instar nymphs. The salivary glands were transferred to a microtube containing 50 μl of 199 Medium (Sigma-Aldrich, St Louis, MO, USA) and then ruptured and homogenized. In each trial, 40 μl of serum (heat or non-heat inactivated) plus 10 μl of a metacyclic trypomastigote suspension (varying between 2 and 3.4 x 10^6^ parasites/ml) were incubated at 41°C or 37°C (with pigeon or mouse sera, respectively) for 30 min. The number of parasites was quantified in a Neubauer chamber at 0 and 30 min of incubation. The percentage of lysis observed in non-heat inactivated serum after 30 min of incubation was normalized in relation to the corresponding values obtained using inactivated serum.

### Statistical analysis

The numbers of parasites present in the salivary glands of fed and unfed insects were compared using the t test. The numbers of parasites in the intestinal tract of nymphs fed on mice at different stages of infection were compared between trials and among days of infection. In this case, data normality was evaluated using Shapiro-Wilk's W. Variables were compared using nonparametric Mann-Whitney and Kruskal-Wallis tests; the groups responsible for the differences found using the Kruskal-Wallis test were identified using the Mann-Whitney test with the Bonferroni correction [31]. Differences were considered significant at p<0.05 except when the Bonferroni correction was used, when differences were considered significant at p<0.005.

## Results

### Estimation of the number of parasites released by *T*. *rangeli*-infected nymphs during a blood meal on mice

The number of parasites/DNA amount present in the salivary glands of unfed and fed *R*. *prolixus* nymphs is shown in the Table 1. Despite the large variability observed among the samples, a significant reduction was observed in the number of parasites/parasite DNA present in the salivary glands after a blood meal (t test, p = 0.001 when counting using a Neubauer chamber and qPCR). The estimations from Neubauer quantifications showed that approximately 54,000 parasites were liberated during a single blood meal, which corresponds to 76% of the parasite burden in the salivary glands of starved nymphs. The relative estimation by qPCR showed that about 68.4% of the parasites inside the glands were released into the host after a blood meal.

**Table 1:** Estimation of the parasite load present in the salivary gland (SG) of starved and fed 5^th^ instar nymphs of *R*. *prolixus* 45 days after infection by *T*. *rangeli* (parasites—Neubauer chamber and RNA-Cl1 sno gene copies/μL—qPCR). Values correspond to means ± SE.

Quantification method	Starved nymphs (n = 11)	Fed nymphs (n = 11)	p value	Parasites released
Neubauer chamber (parasites/SG)	7.1x10^4^±4.0x10^4^	1.7x10^4^±1.4x10^4^	0.001	5.4x10^4^
qPCR (gene copy/SG)	11.4x10^4^±6.4x10^4^	3.6x10^4^±2.7x10^4^	0.001	-

### *T*. *rangeli* transmission from infected mice to *R*. *prolixus* nymphs over time

**Fig 1:**
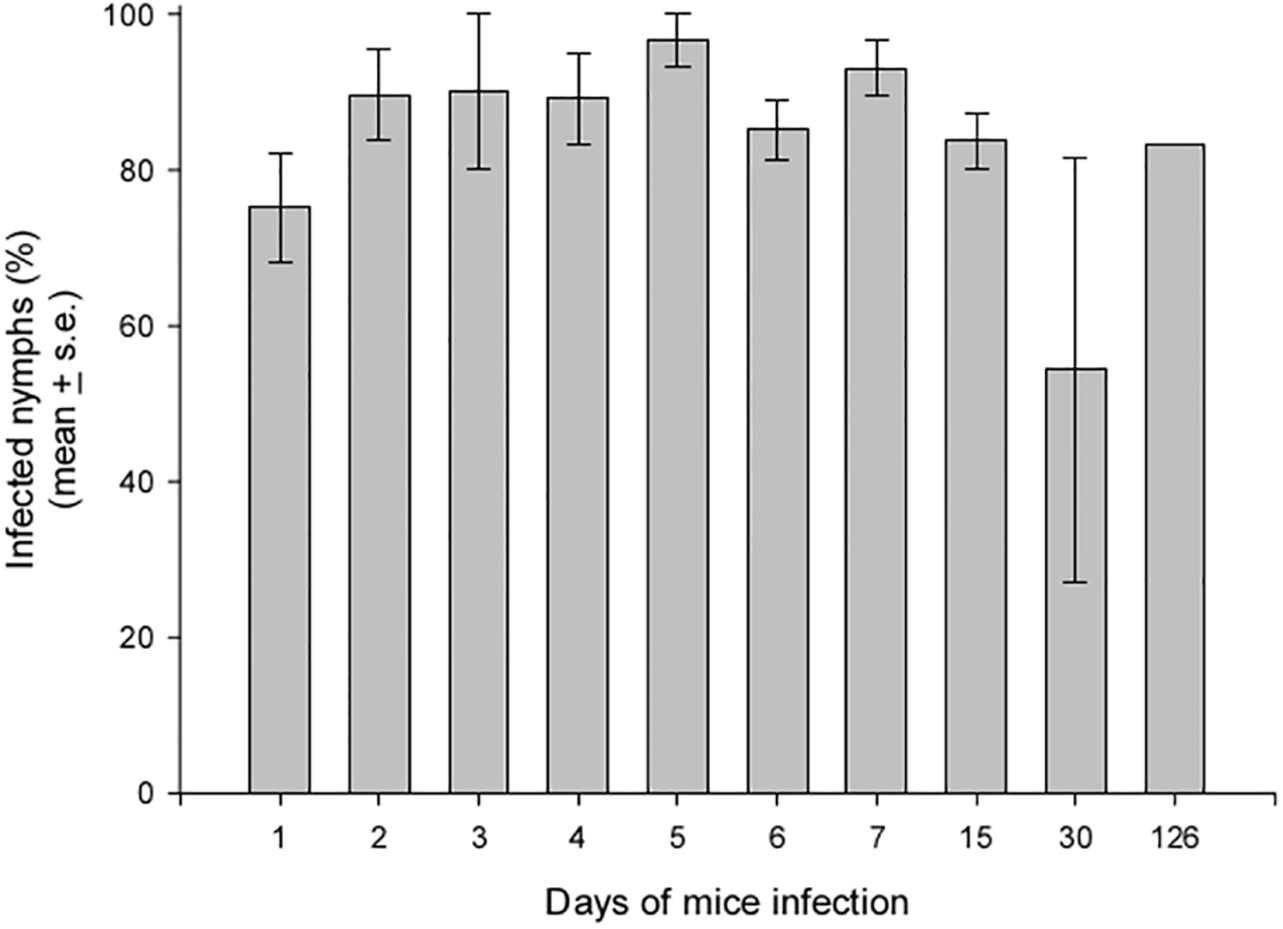
Infection rates of *R*. *prolixus* 5^th^ instar nymphs that fed on *T*. *rangeli-*infected mice on different days after infection. The data shown represent the mean of three biological replicates, except from the data for 126 days post infection, which are based on a single experiment using five tested insects. For the other replicates, 25–30 nymphs were used for each evaluation day.

Twenty-eight (90.3%) of 31 rodents that served as the food source to one infected nymph became infected by *T*. *rangeli*. Infection was confirmed by examining the intestinal contents of fed nymphs and/or hemoculture; parasite transmission to insects was evaluated using these infected mice. The percentage of infected nymphs that fed on mice on different days of infection is shown in Fig 1. Triatomine infection rates were high on all days, reaching approximately 80% except in one replicate, when none of the insects that fed on 30 dpi became infected. Parasites were also present in the hemolymph of 10 infected insects (4.8%), although they were not found in the salivary glands.

**Fig 2:**
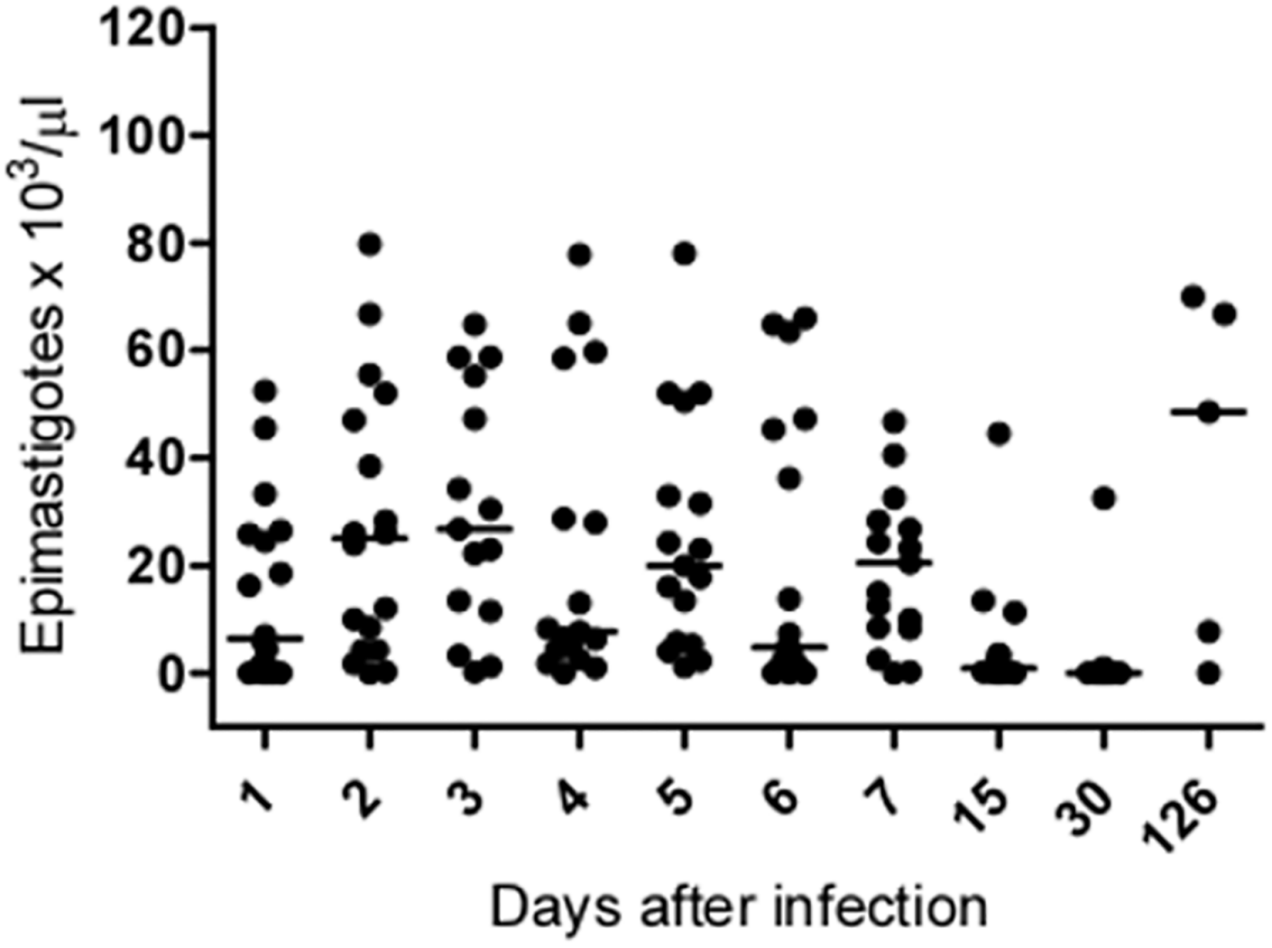
Number of parasites found in the intestinal tract of *T*. *rangeli*-infected *R*. *prolixus* 5^th^ instar nymphs that were fed on mice at different stages of infection. The horizontal lines represent the median value obtained for each group.

The number of parasites found in the intestinal tract of the infected insects was highly variable among samples, but it was possible to observe an effect of the duration of the mouse infection on this parameter (Fig 2, Kruskal-Wallis, p<0.0001). The number of parasites was lower in insects that fed on mice at 15 and 30 dpi, but infection returned to high levels in mice with more prolonged infection (126 dpi).

The number of parasites found in the circulating blood of infected mice was evaluated on 1–7, 15 and 30 dpi. Of the 24 evaluated mice, only two (8.3%; 7 dpi) exhibited *T*. *rangeli* parasitemia.

### Insect-insect *T*. *rangeli* transmission during hemolymphagy

To evaluate the importance of *T*. *rangeli* transmission among insects through hemolymphagy, we developed two assays. Initially, the motivation of starved nymphs to feed on a fed conspecific was evaluated. Only one nymph (5%) of the 20 insects evaluated in two different assays attempted to bite the infected nymph during the assay. In this case, the starved nymph remained in contact with the infected one for 2.7 min with no visual ingestion being observed. Because 10 days of starvation is probably too short a time to trigger feeding in *Rhodnius* nymphs, we decided to keep 2^nd^ instar nymphs, which were starved for 20 days, together with one infected, fed 5^th^ instar nymph for 15 days. The intestinal tracts of the nymphs were examined after the insects had molted to the 3^rd^ instar; however, no insect had been infected.

### Insect-insect *T*. *rangeli* transmission during co-feeding on vertebrate

**Fig 3:**
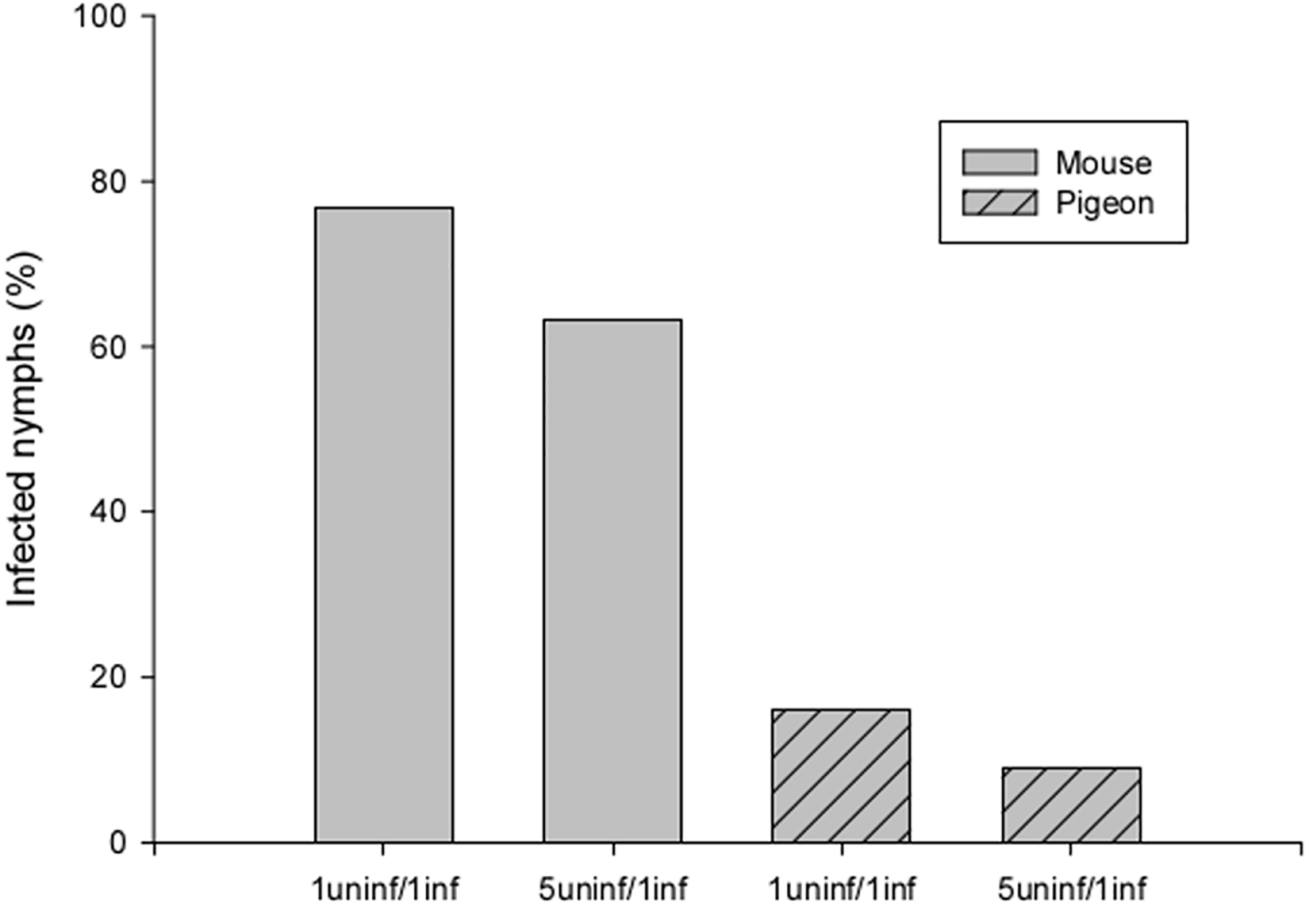
Rates of infection of nymphs that co-fed with *T*. *rangeli-*infected conspecifics (uninf = uninfected; inf = infected). In the 5uninf/1inf assay using pigeons as hosts, recipient insects were 6 cm apart from the donor.

After taking a blood meal on mice together with infected conspecifics, 76.9% of the 5^th^ instar nymphs became infected by *T*. *rangeli* (Fig 3). Even in assays where one donor nymph shared a mammal host with five recipient nymphs, a similar percentage of infection (63.1%) was observed. Interestingly, 16% of the recipient nymphs became infected when pigeons were used as hosts (1 donor *vs* 1 recipient). When one donor together with five recipient nymphs were simultaneously offered a blood meal at two different points on the pigeon, 9% became infected (2 out of 22 individuals, as 3 out of 25 did not feed in the experiments). In the assays in which uninfected nymphs fed on *T*. *rangeli* exposed pigeons either 30 min or 24 h after parasite inoculation, no insects were found to be infected.

### Evaluation of complement-mediated *T*. *rangeli* lysis

**Fig 4:**
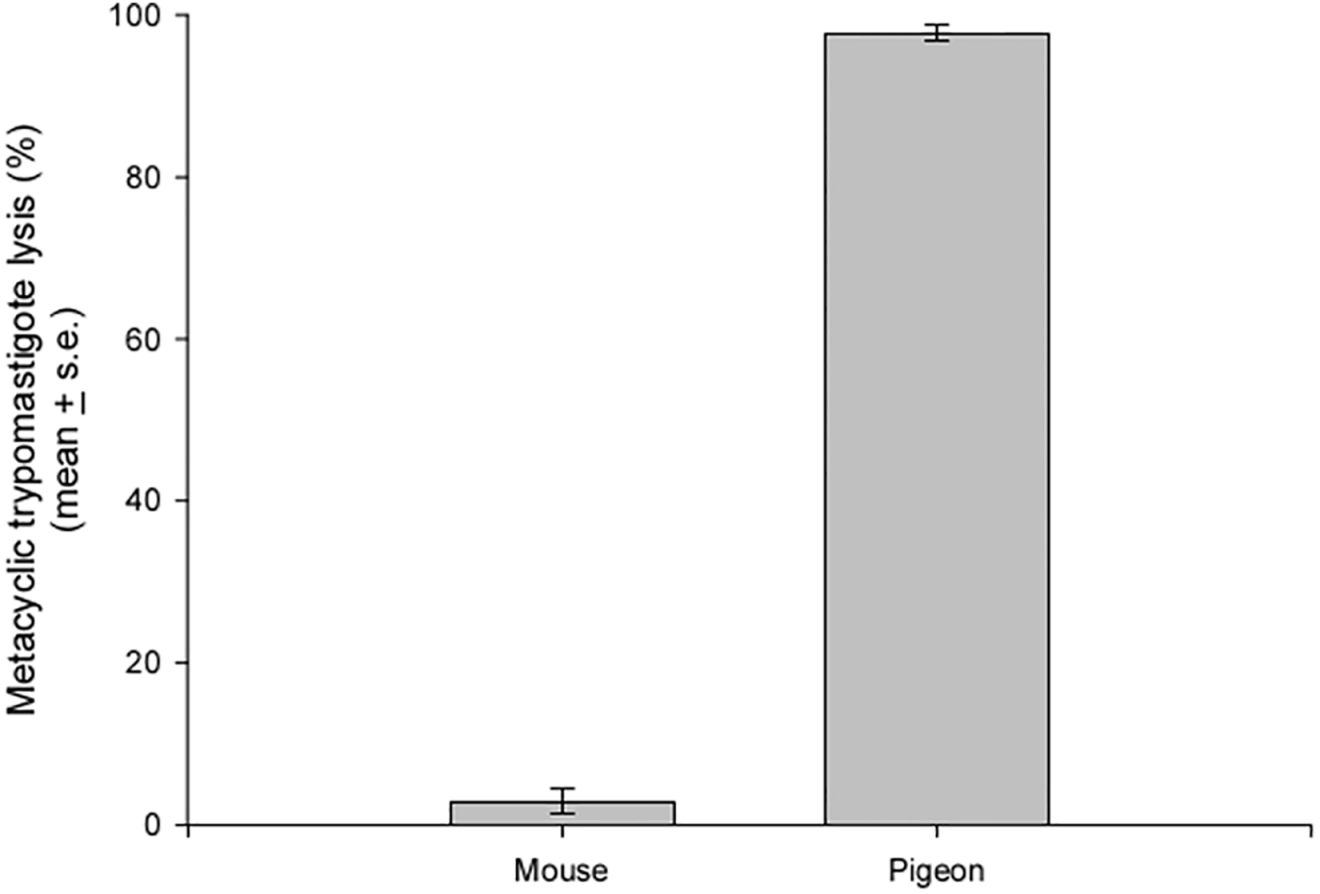
Complement-mediated *T*. *rangeli* metacyclic trypomastigote lysis by mouse and pigeon serum. Data are presented as the percentage of lysed trypomastigotes after 30 min of incubation whit non-heat inactivated serum (n = 3).

To determine whether metacyclic trypomastigotes are lysed by the complement systems of the mouse and pigeon, parasite suspensions that were obtained from infected insect salivary glands were incubated with serum from both hosts. A lysis of approximately 1% was observed in parasites incubated with heat-inactivated sera from both hosts. In the same way, approximately 3% of the parasites were lysed when incubated with non-heat inactivated serum from mice. However, when parasites were incubated with non-heat inactivated serum from pigeons, approximately 98% of the parasites were lysed after 30 min of incubation (Fig 4).

## Discussion

Salivation during feeding is known to occur during both probing and ingestion periods for *R*. *prolixus* [32], and approximately 50% of the saliva which has previously been produced and stored is released during feeding in live host [33]. We have previously shown that an infected *R*. *prolixus* 5^th^ instar can bear up to 100,000 parasites, almost all of them metacyclic trypomastigotes, in its salivary glands [26]. The present study showed that approximately 50,000 parasites can be released during blood feeding. Which proportion of this parasite population is liberated on the skin during probing, directly injected into circulating blood or ingested together with the blood, remains to be determined. Nevertheless, even when a host is inoculated with a large number of parasites, if there are no multiplicative forms present in the vertebrate host, as suggested by several studies [9, 34], the infection will be gradually lost. To evaluate this hypothesis, we infected mice through a single contact with an infected insect and offered these mice to uninfected nymphs on different days after infection. The observed infection rates demonstrated that the transmission of *T*. *rangeli* from insect to mouse is highly efficient, reaching values as high as 90%. In a similar manner, these infected mice were able to transmit the parasite to approximately 80% of the insects that fed on them. Moreover, these elevated infection rates were maintained in insects that fed on mice with old infections, clearly demonstrating that the infection is not lost over time. Given these results, it is likely that *T*. *rangeli* develops a multiplicative cycle in its vertebrate host. The presence of reproductive forms of *T*. *rangeli* in vertebrates is suggested by results obtained in some studies [7, 8, 35]; however, strong evidence of this presence has not been presented. Our results add more support for this idea. In addition, despite the effectiveness of the transmission, parasites were rarely found circulating in mouse blood, even on day 0 when a large number of the parasites had been injected by the infected insect; this finding raises the possibility that the parasites can hide in some host tissues or organs.

Once *T*. *rangeli* colonizes the intestinal tract of the vector, the parasite must cross two important physical barriers, the intestinal epithelium and the salivary glands, to be transmitted to vertebrate hosts. In the present work, approximately 5% of nymphs that presented parasites in the intestinal tract also showed parasites in the hemolymph. The number of parasites found in the intestinal tract of these insects depended on the age of the mouse infection. Interestingly, mice with older infections produced infections with similar numbers of parasites to those of recently infected mice. Based on these results, we speculate that the parasite might modify its capacity to infect insects over time due to development in the vertebrate host. Nevertheless, the number of parasites present in the insect gut appears unrelated to hemolymph invasion because this apparently occurred at random. The ability to reach the hemolymph appears to serve as a critical filter because hemolymph infections can occur at frequencies from 2 to 50% in insects with parasites in the intestinal tract [25, 36–38]. Perimicrovillar membranes have been implicated in the invasion process because insect exposure to radiation affects the organization and structure of microvilli, thereby allowing the parasites to reach the hemolymph earlier than in insects that are not exposed to radiation [39]. No parasites were found inside the insect salivary glands at two months post infection. Possibly, this period might be too short to allow the parasite to complete its development in the infection conditions we used. In infections in which the hemolymph was directly inoculated with the parasites from the same strain, a nine-day delay was observed before the parasites began to invade the salivary glands [26].

Because a small proportion of insects harboring parasites in the intestinal tract will also present infective parasites in the salivary glands, an effective transmission strategy is crucial for the efficient spread of the parasite to both vertebrate and triatomine hosts. Under our experimental conditions, *R*. *prolixus* nymphs did not exhibit motivation to feed on conspecifics. Because *R*. *prolixus* can survive for up to three months without food [40], we increased the starvation status in experimental nymphs and allowed them to remain with infected, fed nymphs for a longer period. However, prolonged nutritional stress associated with an increase in the period of exposure to infected nymphs did not promote parasite transmission. Hemolymphagy has been described for several triatomine species [29, 41] including *R*. *prolixus* [21, 42], and the occurrence of this behavior has been associated with nutritional stress at the early nymphal stages [21, 29]. Transmission of *T*. *rangeli* through hemolymphagy has been reported [21] when *R*. *prolixus* 1^st^ instar nymphs were infected after remaining together with *R*. *prolixus-* and *Rhodnius robustus*-infected 5^th^ instar nymphs. Nevertheless, another study did not find transmission when 1,000 *R*. *prolixus* nymphs were exposed to infected insects for 15 days [43]. Under the conditions evaluated in the present study, the transmission of *T*. *rangeli* between nymphs through hemolymphagy would not play a relevant role in parasite circulation. Even so, it would be relevant to evaluate the possibility of insect-insect *T*. *rangeli* transmission in other contexts, such as through hemolymphagy during the molting period or in instances of coprophagy or cleptohematophagy (the taking of blood from recently fed conspecifics, as often observed in laboratory feeds).

An alternative mode of *T*. *rangeli* transmission is parasite transference from an infected to an uninfected insect when the insects feed simultaneously on the same vertebrate host. The co-feeding transmission using mammals as hosts has been previously reported in triatomines [22], although the experimental conditions were not clearly described. In the present study, we used a device to physically separate infected and uninfected nymphs to prevent cleptohematophagy. Under these conditions, a high transmission rate was achieved. Even when the infected nymph was allowed to feed with five uninfected nymphs, the transmission rate was still elevated. Therefore, our data strongly suggest that this mode of transmission can play a relevant role in the maintenance of *T*. *rangeli* in nature in the absence of a host with systemic infection. The efficiency of pathogen transmission by co-feeding in ticks has been demonstrated for several tick-borne viruses and bacteria (see revision in [44]). Virus transmission was supported even when natural hosts having neutralizing antibodies were used as hosts [45]. Co-feeding transmission of West Nile virus has been showed to occur in argasid tick species, which unlike ixodid ticks, typically feed for less than 2 hours [46]. In addition, the vesicular stomatitis virus has been showed to be transmitted by black flies during co-feeding in non viremic hosts [47].

Unexpectedly, when pigeons were used as a blood source, 16% of the healthy nymphs that had co-fed with one infected conspecific became infected. Parasites were transmitted even when uninfected and infected nymphs were placed at distant points on the host. It is worth mentioning that this represents the first time that a bird has been implicated in the transmission of a trypanosome from the *T*. *cruzi* clade. The refractory conditions of avian blood for trypanosomes of this clade is well known because the infectious forms are lysed by the avian complement system [48, 49], which plays an important role in innate immunity by acting as a first barrier against pathogens. Cultured epimastigotes of *T*. *rangeli* have been shown to be resistant to complement-mediated lysis in several mammal species [16, 50, 51]. These *T*. *rangeli* forms, however, are lysed by normal fresh chicken serum [50]. To our knowledge, no studies have evaluated the susceptibility of *T*. *rangeli* metacyclic trypomastigotes to a complement system; therefore, we incubated these forms with non-heat inactivated sera from mouse and pigeon. As seen with epimastigotes, metacyclic trypomastigotes were lysed by pigeon but not by mouse serum. Nevertheless, our results showed insect-insect transmission during co-feeding when pigeons were used as hosts, even if in a lower rate than those observed when mice were the living hosts. This surprising finding suggests the possibility that among the thousands of parasites liberated during the blood ingestion, some can reach the receiving insect before the action of complement. Once inside the insect intestinal tract, parasites would be protected against complement action because both the saliva and the intestinal contents of triatomines contain complement inhibitors that protect the intestinal epithelium from damage [52].

Triatomine insects are opportunistic feeders that are adapted to occasional, large meals. These insects live in small colonies in association with warm-blooded animals in nests that are usually temporarily occupied and that depend on the food supply in the vicinity and offspring growth. Feeding motivation, as several other behaviors expressed by triatomines, is temporally-modulated, being the individuals more likely to feed during the first hours of the night (see revision in [53]). In this sense, one can expect that co-feeding is a behavior commonly found in the natural refugees of these insects. Our results indicate that the presence of a vertebrate, independently of being infected, and even if transient, would ensure parasite spreading among individuals in a colony. Since *Rhodnius* species can be found associated with avian nests [54, 55] the finding that birds can also allow parasite transfer between insects opens a new avenue of ecological and epidemiological studies on *T*. *rangeli* transmission.

## Conclusions

Our results showed that *T*. *rangeli* is easily acquired by triatomines during blood feeding on mice. Even if no parasitemia is apparent, these infected mammals can act as sources of *T*. *rangeli* for long periods. *T*. *rangeli* was also efficiently transmitted from insect to insect by co-feeding on mice. In addition, we showed for the first time that transmission by co-feeding on pigeons is also feasible. Based on our results, we suggest that both, efficient parasite acquisition during blood feeding on infected mammals and the process of infection through co-feeding, would ensure a high number of insects harboring parasites in their intestinal tracts. Due to the variable (sometimes low) rate of parasites crossing the intestinal epithelium a higher number of gut infected insects would mean that a higher percentage of them present parasites in the salivary glands, therefore increasing the probabilities of transmission to mammal hosts. Finally, efficient transmission by inoculation of infective forms to mammal hosts would grant cycle completion.
